# Theoretical study of time-resolved photoelectron circular dichroism in the photodissociation of a chiral molecule

**DOI:** 10.1063/4.0000213

**Published:** 2023-12-15

**Authors:** Marit R. Fiechter, Vít Svoboda, Hans Jakob Wörner

**Affiliations:** Laboratory of Physical Chemistry, ETH Zürich, 8093 Zurich, Switzerland

## Abstract

Photoelectron circular dichroism (PECD), the forward–backward asymmetry of the photoelectron angular distribution when ionizing randomly oriented chiral molecules with circularly polarized light, is an established method to investigate chiral properties of molecules in their electronic ground state. Here, we develop a computational strategy for predicting time-resolved PECD (TRPECD) of chemical reactions and demonstrate the method on the photodissociation of 1-iodo-2-methylbutane. Our approach combines multi-configurational quantum-chemical calculations of the relevant potential-energy surfaces of the neutral and singly ionized molecule with *ab initio* molecular-dynamics (AIMD) calculations. The PECD parameters along the AIMD trajectories are calculated with the aid of electron-molecule scattering calculations based on the Schwinger variational principle implemented in ePolyScat. Our calculations have been performed for two probe wavelengths (133 and 160 nm) accessible through low-order harmonic generation in gases. Our results show that the TRPECD is a highly sensitive probe of photochemical reaction dynamics. Most interestingly, the TRPECD is found to change sign multiple times along the photodissociation coordinate, in agreement with recent experiments on CHBrFI [Svoboda *et al.*, “Femtosecond photoelectron circular dichroism of chemical reactions,” Sci. Adv. **8**, eabq2811 (2022)]. The computational protocol introduced in the present work is general and readily applicable to other chiral photochemical processes.

## I. INTRODUCTION

Chirality, the non-superimposability of the mirror image of an object with the object itself, is an ubiquitous phenomenon in the world we live in—ranging from the macroscopic level with the handedness of snail houses, which are preferentially left-handed with a ratio of 20 000:1,^1–3^ to neutrinos on the level of elementary particles. These particles are known to only occur in a left-handed form, while their antiparticles are exclusively right-handed.^4^ The concept of chirality is of fundamental importance in chemistry and biochemistry as well. Of each chiral molecule, there exist two mirror images, commonly referred to as enantiomers. The importance of chirality in biochemistry is for a large part due to so-called “biomolecular homochirality,” which in turn has a large pharmacological impact.

Since enantiomers are structurally identical up to a parity transformation, most spectroscopic techniques are not able to differentiate between them. The only way to spectroscopically acquire sensitivity to the handedness of a chiral molecule is by using a chiral reference: left or right circularly polarized light (LCP and RCP, respectively). This type of method is commonly referred to as chiroptical. One often-used chiroptical technique is circular dichroism (CD), in which differences between the absorption of LCP and RCP light are measured. However, since the helical pitch of circularly polarized light in the visible/UV range is much larger than the extent of a small molecule, the molecule effectively does not “feel” much of the chirality of the incident light. This is why the absorption CD is typically very weak,^5,6^ corresponding to relative signal-intensity changes on the order of $10^{-6}-10^{-3}$ between the two enantiomers.^7^ Much larger CDs can be obtained in the x-ray domain, where predictions of time-resolved CD measurements are promising.^8,9^

Improving on methods for differentiation between enantiomers and absolute configuration determination forms an active field of research. In recent years, few new methods have been demonstrated, including Coulomb explosion imaging,^10–12^ a three-wave mixing scheme in microwave spectroscopy,^13–15^ and high-harmonic spectroscopy (HHS) in weakly elliptical^16^ and bi-circular laser fields.^17–19^ Additionally, another chiroptical method has emerged: photoelectron circular dichroism (PECD).^20–22^ This effect is observed as the forward/backward asymmetry of an electron emitted by a chiral molecule with respect to the light propagation axis, upon ionization with circularly polarized light. The asymmetry can be orders of magnitude larger than conventional circular dichroism since it is allowed within the electric dipole approximation; asymmetries of up to several tens of percent have been observed, e.g., 25% in methyl *p*-tolyl sulfoxide,^23^ motivating the present work.

Static chiral experiments are currently fairly well established; chirality is, however, a relatively unexplored area within the field of ultrafast dynamics. For the femtosecond pump-probe experiments unique to this field, a feasible method has to meet a few criteria: first, if we want to study the chiral dynamics of the isolated molecule, implying a gas-phase measurement, CD is a more challenging approach as its signal is so weak. Second, both pump and probe have to be femtosecond pulses, ruling out microwave spectroscopy. Additionally, Coulomb explosion imaging is somewhat limited in its ability to follow chiral dynamics, as typically four fragments have to be detected in coincidence in order to determine the absolute configuration. The long integration times required for this technique make time-resolved measurements time consuming.

This leaves HHS and PECD as key table-top techniques for chiral discrimination on femtosecond timescales. Both methods have very recently been applied to the real-time probing of chirality during chemical reactions.^19,24^ In these time-resolved PECD (TRPECD) experiments, 2-iodobutane and CHBrFI were photoexcited to dissociative states by a linearly polarized pump pulse, and the dissociation reaction was followed by photoionization with a fully characterized circular probe pulse.^25^ The angular distribution of the ejected photoelectrons was recorded with a position-sensitive detector to yield the TRPECD signal. This type of experiment can reveal details of the time evolution of chirality during a chemical reaction, which is fundamental to understanding the aforementioned chiral recognition mechanisms in biochemistry. Related TRPECD experiments have also been performed on the Coulomb explosion of doubly charged trifluoromethyloxirane following F(1s) core-shell ionization.^26^

This work presents a complete protocol for the theoretical prediction of TRPECD during photochemical processes. In general, PECD effects are calculated by first solving the electron-molecule scattering problem, i.e., finding the bound and continuum wavefunctions. Using these, the photoionization matrix elements can be calculated, from which the PECD can be extracted.^27^ There are two well-established methods to predict PECD: the CMS-X*α*^27^ and B-spline^28,29^ methods. In this work, we present a complementary approach to calculate PECD based on the variational Schwinger method and a single-center expansion as implemented in ePolyScat.^30,31^ First results using this approach have been presented in Refs. 23 and 24. In contrast to the previously used CMS-X*α* and B-spline methods, which use the Hamiltonian formulation of the quantum-scattering problem, ePolyScat uses the Lippmann–Schwinger equations. In addition to being applicable to PECD calculations of static molecules, our method is also applied to describe time-dependent chirality changes over the course of a photochemical reaction. This generally applicable approach for calculating TRPECD is illustrated on 1-iodo-2-methylbutane, which has recently been experimentally studied at FLASH,^32^ but can readily be generalized to describe other chiral photochemical processes.

## II. COMPUTATIONAL METHODS

### A. Potential energy surfaces along the C–I bond

All calculations in this section were performed using the quantum chemistry package ORCA.^33^ To start with, the geometry of 1-iodo-2-methylbutane was optimized on the MP2 level with the Karlsruhe def2-TZVP (triple-zeta-valence-polarization) basis set,^34^ which comes with a Stuttgart–Dresden effective-core potential for iodine.^35^ Unless otherwise mentioned, all calculations were performed on the most stable conformer (“conformer 1”) of the molecule. The role of other conformers is discussed in Sec. III E. Subsequent scans of the potential-energy surfaces along the C–I bond were performed on the complete-active-space self-consistent-field (CASSCF) level, starting from the MP2 orbitals at the equilibrium geometry and then progressively stretching the C–I bond until dissociation. This serves as a quick check before starting more expensive calculations and yields an orbital guess at the dissociation limit. After this, we performed a QD-NEVPT2 calculation (quasi-degenerate N-electron valence perturbation theory), which adds a perturbative correction to a CASSCF wave function adapted to avoid artifacts arising from states approaching each other closely in energy. With this we scanned inwards along the C–I bond. For both scans, we used a chemically motivated (6,4) active space, because we expected the three 5*p*-orbitals on iodine and one *sp*^3^-hybridized orbital on the neighboring carbon atom to be most important for describing the dissociation. This assumption was theoretically verified by a MP2 calculation providing partial occupation numbers for the involved orbitals in the range between 0.05 and 1.95. Moreover, these orbitals were subjected to intrinsic bond orbital (IBO) analysis confirming their character as lone pair orbitals on the iodine atom and *σ* bonding and anti-bonding orbitals of the C–I bond. All of these CASSCF-based calculations were state-averaged, containing three singlet and three triplet states with equal weightings.

For the cation, we did not use a reoptimized geometry, as it is not much different from the geometry of the neutral molecule, and on the timescales of the experiment, structural relaxation is unlikely to fully take place. We performed a MRCI calculation in ORCA including 12 doublet and three quadruplet states and using the same basis set as before.

### B. *Ab initio* molecular dynamics

The *ab-initio* molecular-dynamics (AIMD) calculations were performed in Q-Chem.^36^ The ground-state geometry was first optimized at the density-function theory (DFT) level with a B3LYP functional and the 6-311 G(d,p) basis set. As the optimized ground-state geometry was used to sample the excited-state starting geometries and nuclear velocities from, we employed a Langevin thermostat as implemented in Q-Chem to ensure that we sampled from a canonical distribution.^37^ Vertical impulsive photoexcitation was assumed in the AIMD calculations, but the effect of finite pulse durations was included later, as described in Sec. II C.

The excited-state AIMD calculations used the same basis set but were run at the configuration interaction singles (CIS) level of theory, including the first three singlet excited states; the AIMD trajectories were propagated on the lowest of these surfaces. From the calculated dynamics, we can extract the dissociation time, which we define here as being the time required to reach twice the C–I equilibrium bond distance. We have verified that potential-energy surfaces calculated at the same level of theory using the same basis set display the correct dissociation behavior.

### C. Time-resolved photoelectron spectra

For calculating the time-resolved photoelectron spectra (TRPES), we assume 266-nm pump, and 133- or 160-nm probe pulses, as these are directly available from low-order harmonic generation in gases with both linear^38–40^ and circular polarizations.^23–25,41^ The pump pulse will excite the molecule from its electronic ground state to the lowest-lying absorption band. From here, it will evolve on this surface (where our assumption is that the internal motion of the molecule primarily involves the C–I coordinate, we use the potential surfaces in Subsection II A here). When the probe pulse comes in after some time delay, the molecule is excited to a cationic surface, i.e., ionized. The photoelectron kinetic energy (PKE) at a certain point along the C–I dissociation curve can be found from the difference between the probe energy and the local binding energy. Using the bond-length-to-time mapping we obtained from the AIMD calculations, we can convert the C–I distance to the pump-probe delay, so that we obtain the photoelectron kinetic energy as a function of time.

Except for the desired signal (photoionization of the first excited state), there are a few other sources of photoelectrons that we expect to see in our spectra. For instance, direct ionization of the ground state may play a role and contribute to a background of low-kinetic-energy photoelectrons. Also, we expect to see a time-zero feature, due to two-photon-ionization processes induced by one pump and one probe photon.

Having included these sources of photoelectrons, the only task left is now to incorporate the finite spectral and temporal resolution in the predicted spectra, which was done by convoluting the photoelectron kinetic energy curve with Gaussian-profile functions of chosen width. For the energy convolution, we chose a full-width at half maximum (FWHM) of 0.4 eV, which is a conservative estimate based on previous related experiments.^38–40^ The dominant factor in this case is the spectrometer resolution. The temporal resolution is varied in order to investigate the effect of the pulse width on the information content of the time-resolved spectra.

### D. Time-resolved photoelectron circular dichroism

The electron scattering calculations were performed using the ePolyScat quantum-scattering package.^30,31^ This code solves the quantum-mechanical electron-molecule scattering problem with a single-center partial-wave expansion using the Schwinger variational principle and returns the photoionization matrix elements. From these matrix elements, we can extract *b_i_* coefficients by following the same approach as was done in the supplementary material of Svoboda *et al.*^24^ As shown by Powis,^22^ these coefficients enter the traditional expansion of the photoelectron angular distribution in a Legendre-polynomial series

$$I_{p}(\theta )=1+b_{1}^{\left\{p\right\}}P_{1}(\text{cos}\;\theta )+b_{2}^{\left\{p\right\}}P_{2}(\text{cos}\;\theta ),$$ (1)

where *θ* corresponds to the electron ejection angle with respect to the light propagation direction, *p* stands for the polarization of light (0 for linear, ±1 for left/right circular polarization), and *P*_1_ and *P*_2_ are the first and second Legendre polynomial, respectively. In this way, we obtain the values of *b*_1_ and *b*_2_, which quantify the PECD as

$$\text{PECD}=2\frac{b_{1}\;\text{cos}\;\theta }{1+b_{2}[0.5(3{\;\text{cos}\;}^{2}\theta -1)]}.$$ (2)

This equation can be further simplified by taking only the PECD values along the light-propagation direction to get PECD ( $\theta =0{}^{\circ})=2b_{1}/(1+b_{2})$.

The ePolyScat program needs input orbitals for the excited state as an input for the calculation of the excited-state photoionization matrix elements. These were obtained using the MOLPRO package,^42^ with a state-specific CASSCF approach using a (2,2) active space (i.e., two electrons in the highest-occupied and the lowest-unoccupied molecular orbitals) with a unit weight given to the first electronically excited state. This approach is tailored to achieve the best possible representation of the electronically excited state within the limitations imposed by ePolyScat, i.e., integer occupation numbers of the individual orbitals. In the MOLPRO calculations, we used the correlation-consistent polarization-valence-triple zeta (cc-pVTZ) basis sets for carbon and hydrogen,^43^ and an all-electron (augmented) aug-cc-pVTZ basis for iodine.

A particularly important aspect for PECD calculations is the convergence of the electron-molecule scattering calculations, especially when a single-center expansion is used. Therefore, in the present work, we performed detailed convergence studies and found that *b*_1_ tends to converge much more slowly with the size of the angular-momentum basis, compared to *b*_2_. The two key parameters to converge these calculations are LMax, the maximum angular momentum quantum number ℓ used in the expansion of the wave functions, and LMaxI, used for the numerical grid. We varied both parameters up to LMax = 90 and LMaxI = 220 and found that LMax = 70 and LMaxI = 200 yield converged PECD parameters for the present problem. These values were used in all PECD calculations reported herein.

We performed the calculation of photoionization matrix elements and the extraction of *b*_1_ and *b*_2_ coefficients for eight different photoelectron energies with structures taken from the AIMD simulations, at time intervals of at most 10 fs, but less if we noticed the *b*_1_ or *b*_2_ parameters to vary strongly over time in a particular interval. In other words, the calculations of the photoelectron asymmetry parameter were done for an average molecular structure corresponding to a given pump-probe delay. This yields a map of the PECD parameter as a function of photoelectron kinetic energy and time after excitation.

Not all of the photoelectron energies at all of the delay times are relevant to our problem. In fact, as described in Sec. II C, we have a relation between photoelectron kinetic energy and the delay time in the form of the time-resolved photoelectron spectrum: $E_{PE}(t)$. This relation defines a path through the PECD map that we have created.

**FIG. 1.**
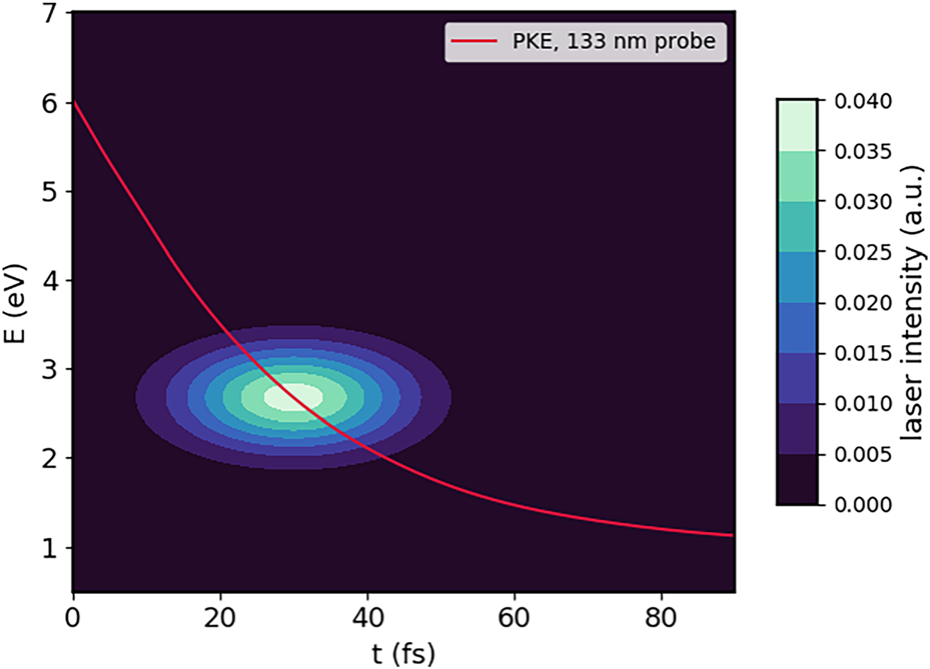
The shape of the laser pulse in energy and time at delay time *t* = 30 fs and with a cross-correlation time of 25 fs FWHM.

We could extract the PECD parameter as a function of delay time by assigning it to be the value at time *t* and photoelectron kinetic energy $E_{PE}(t)$. However, the pump and probe laser pulses are far from being delta functions in energy and time; therefore, the path will be slightly blurred in both directions and regions of the PECD map right next to the path (at lower/higher energies and earlier/later times) will also contribute. In order to account for this, we modeled the laser pulse at delay time *t* by a normalized Gaussian in energy and time, centered at *t* and $E_{PE}(t)$ (see Fig. 1). For the Gaussian in energy, we chose the FWHM to be 0.4 eV, as before, and we again varied the FWHM in time to investigate the influence of the temporal resolution on the magnitude of the observable PECDs. We multiplied this normalized Gaussian centered at delay time *t* point-by-point with the PECD map. The final value of the PECD at this delay time was obtained by integrating over this part of the PECD map.

## III. RESULTS AND DISCUSSION

### A. Potential energy surfaces along the C–I bond

**FIG. 2.**
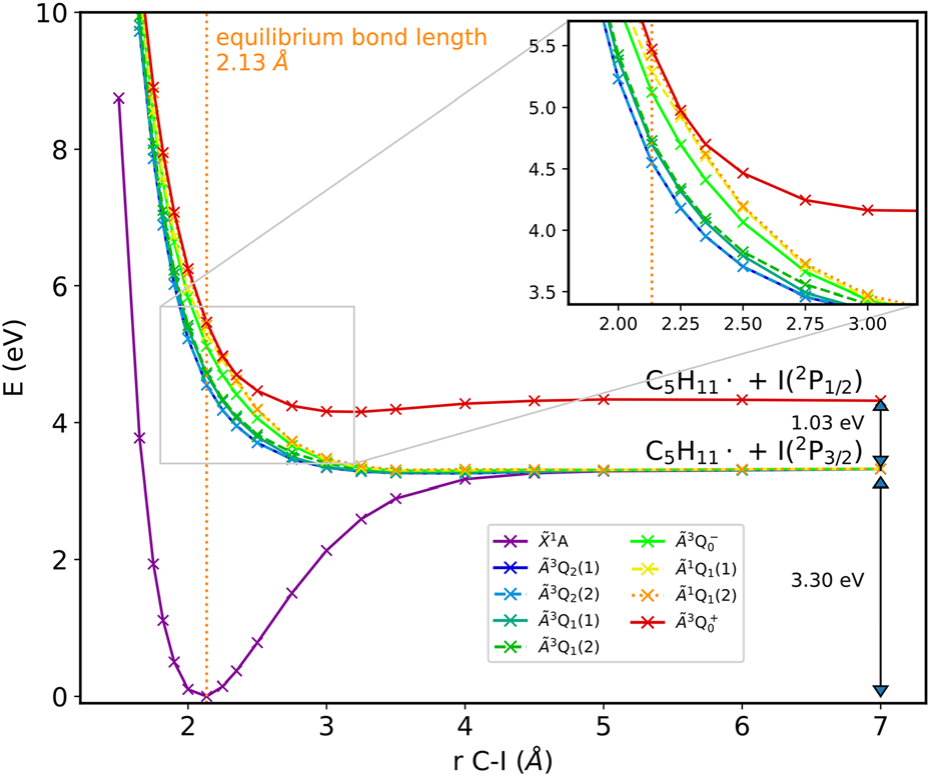
Potential-energy curves as a function of the C–I bond length of neutral 1-iodo-2-methylbutane, calculated using QDNEVPT2 with a (6,4) active space. The states are labeled according to their character after the conical intersection at 2.25 Å; at shorter bond lengths, the labels ${\tilde{A}}^{1}Q_{1}(1)$ and ${\tilde{A}}^{3}Q_{0}^{+}$ should be interchanged. For further details see text.

**FIG. 3.**
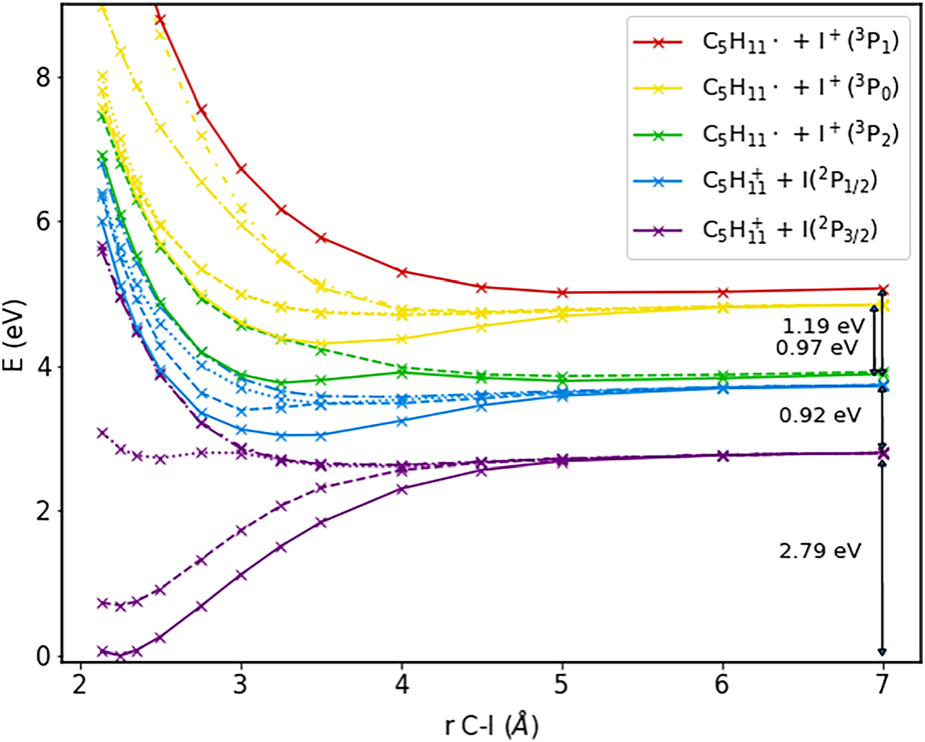
Potential-energy curves as a function of the C–I bond length of cationic 1-iodo-2-methylbutane, calculated with CISD(T) with a CASSCF(5,4)-wavefunction as a reference. For further details, see text.

The first step toward the photoelectron spectrum of 1-iodo-2-methylbutane consists in calculations of the potential-energy curves for the neutral and cationic molecule; these curves are displayed in Figs. 2 and 3.

The states of the neutral molecule are labeled by Mulliken's $C_{3v}$ symbols,^44^ in analogy to CH_3_I. Note that since 1-iodo-2-methylbutane is not actually $C_{3v}$ symmetric, the ${\tilde{A}}^{3}Q_{2},\;{\tilde{A}}^{3}Q_{1}$, and ${\tilde{A}}^{1}Q_{1}$ states are not perfectly degenerate anymore; we have therefore labeled the resulting states within each pair with (1) and (2). In the dissociation limit, we observe two distinct states due to the spin–orbit coupling of iodine. The obtained splitting of 1.03 eV agrees well with the spin–orbit splitting of atomic iodine of 0.94 eV as listed in the NIST spectral database.^45^ In the inset in Fig. 2, it can be seen that there is a conical intersection. As we will see later, this influences the time-resolved photoelectron spectra, as it allows for population to cross from a state that ends up in the lower dissociation limit to a state corresponding to the upper dissociation limit, thereby influencing the photoelectron kinetic energy. The cationic states in Fig. 3 are labeled according to their dissociation limit. Also here, the states at that limit assigned to the cationic molecular fragment and neutral iodine atom exhibit a splitting that is in very good agreement with the spin–orbit splitting of iodine according to the NIST spectral database. The other three states at the dissociation limit correspond to the radical molecular fragment and cationic iodine; for the splitting observed here, the quantitative agreement with the spin–orbit splitting of cationic iodine (0.80 and 0.88 eV^45^) is not as good, but the difference is only about 0.3 eV, and the pattern matches. Additionally, independent calculations on the iodine atom and cation as well as the corresponding radicals agree favorably with this identification of the dissociation limit, confirming its assignment.

### B. *Ab initio* molecular dynamics

**FIG. 4.**
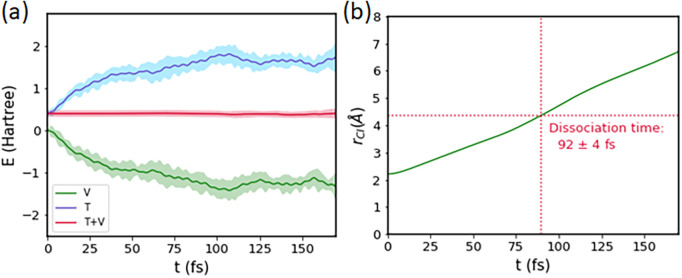
Summarized results of the average over 17 excited-state AIMD simulations. (a) The potential, kinetic, and total energies over the course of the AIMD. (b) Average C–I internuclear separation as a function of time. All errors indicated are calculated from the Student's t-distribution for a two-sided 95% confidence interval.

Next, we performed AIMD simulations, in order to obtain a mapping from C–I bond distance to pump-probe delay time. In combination with the potential-energy curves discussed in Sec. III A, this provided the time-resolved photoelectron spectrum. The starting configuration and nuclear velocities were sampled from the thermal ground-state AIMD. The results of the excited-state AIMD are shown in Fig. 4, which shows an average over all 17 excited-state AIMD trajectories. As can be seen in panel (a), the total energy remains constant over the course of the AIMD simulations, as it should. In panel (b), we show the carbon–iodine bond distance over time. From this, we extract the dissociation time of 92 ± 4 fs, defined as the time required by the C–I internuclear separation to reach twice the equilibrium bond length of the ground-state molecule. We can also obtain a bond-length-to-time mapping from this plot with a linear fit to the curve, yielding a value of 0.0265 Å/fs. We have used this information below in the TRPES prediction to convert the potential energy vs. C–I distance curves we have obtained in Sec. III to potential-energy vs. delay time curves.

**FIG. 5.**
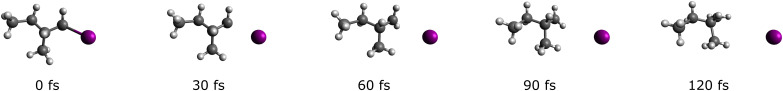
Snapshots of the structure of the dissociating molecule, over the course of one of the AIMD trajectories. The iodine, hydrogen, and carbon atoms are shown in purple, white, and gray, respectively.

In Fig. 5, a few snapshots of one of the excited-state AIMD trajectories are shown, neatly displaying the dissociation of iodine. In our simulations, the molecular fragment retains chirality, and does not spontaneously invert, i.e., it does not stereomutate.

### C. Time-resolved photoelectron spectra

**FIG. 6.**
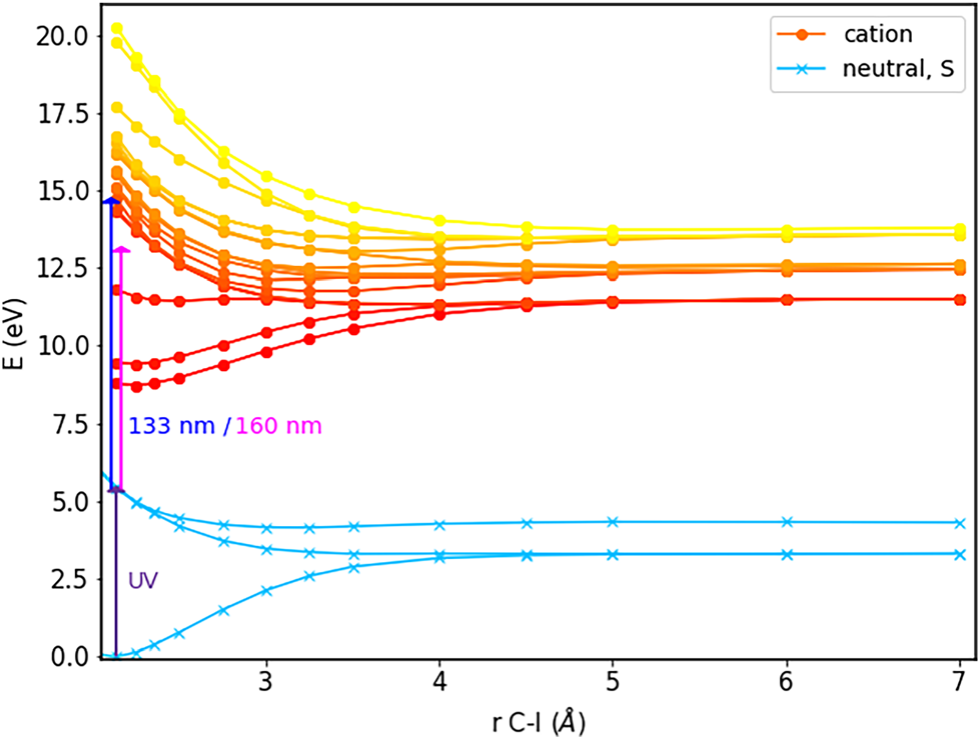
The singlet states in the neutral molecule together with the cationic states mapped out along the dissociation coordinate.

Figure 6 shows both the singlet states of the neutral molecule and the states of the cation as a function of the dissociation coordinate. Since the surfaces have been calculated at a different level of theory, we calculated the ionization potential at equilibrium from comparison of the ground state of a QD-NEVPT2 calculation of the cation with that of the neutral molecule. We use this ionization potential to fix the vertical separation between the neutral QD-NEVPT2 curves and the cationic MRCI curves at the equilibrium geometry.

**FIG. 7.**
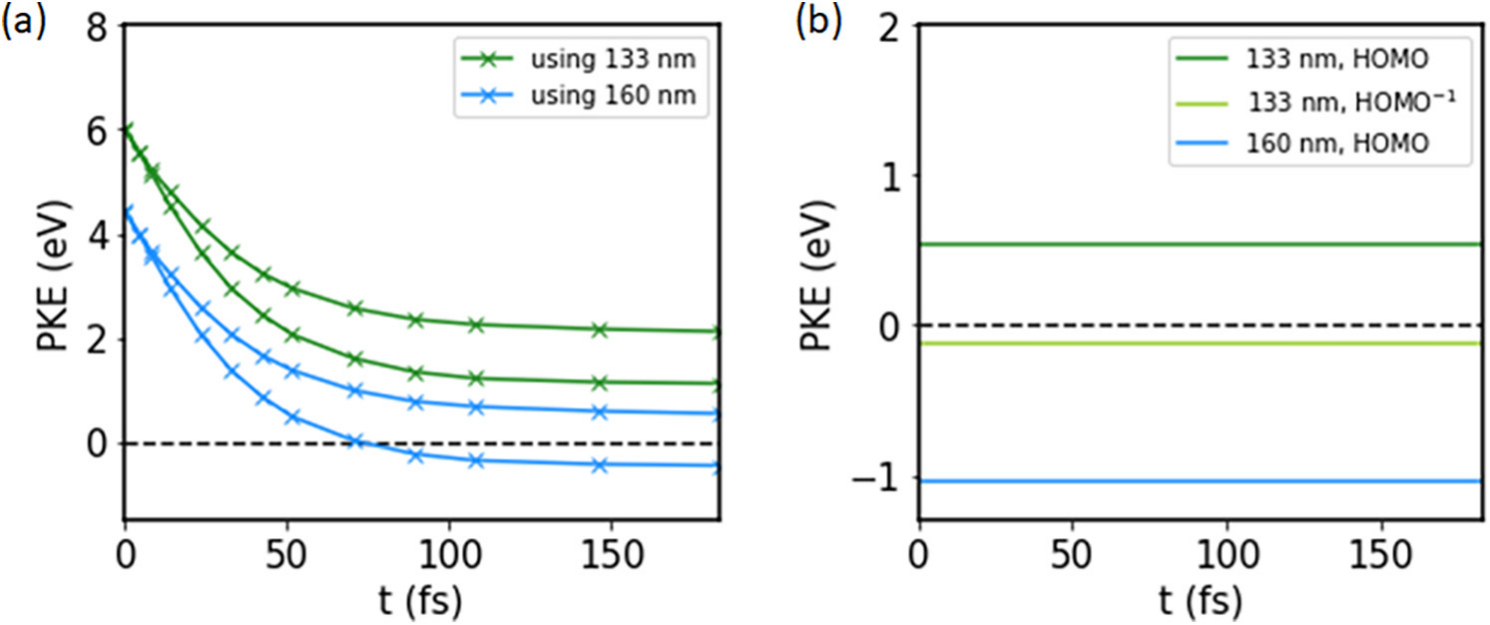
Photoelectron kinetic energies as function of pump-probe delay in the case of (a) ionization of the first excited state and (b) direct ionization of the ground state with the probe, which is time-independent. In both cases, only excitation to the ground state of the cation is considered. In (a), two curves per wavelength are shown due to a possible non-adiabatic transition at the conical intersection from lower to higher spin–orbit state in the neutral molecule.

Also indicated in Fig. 6 are the energies corresponding to the wavelengths of 160 and 133 nm accessible to low-order harmonic generation in gases. The excess energy supplied upon transition from the neutral to the cationic state will be translated into the kinetic energy of the emitted electron. For our molecule, this photoelectron kinetic energy is shown in Fig. 7 as a function of the C–I bond length. Because of a conical intersection of two surfaces in the neutral molecule, some population may crossover to occupy the higher-lying spin–orbit state in the dissociation limit. This is why for a single probe wavelength and excitation only to the cationic ground state, two photoelectron kinetic energy (PKE) curves are shown in the left panel. Furthermore, judging by both (a) and (b), the choice between a 133 nm or a 160 nm probe will have considerable consequences for the experiment. From (a), it is clear that with 133 nm, we can follow the entire decay of PKE to a fixed value over time, whereas when using 160 nm, we can only follow the upper spin–orbit state. For the lower spin–orbit state, the vertical ionization energy will exceed the photon energy when the C–I bond is stretched to beyond roughly 4 Å. This in itself is not a major limitation since we expect the interesting behavior to have occurred before that. Additionally, using the 160-nm probe wavelength comes with the advantage of a negligible background from direct ionization, as shown in panel (b).

**FIG. 8.**
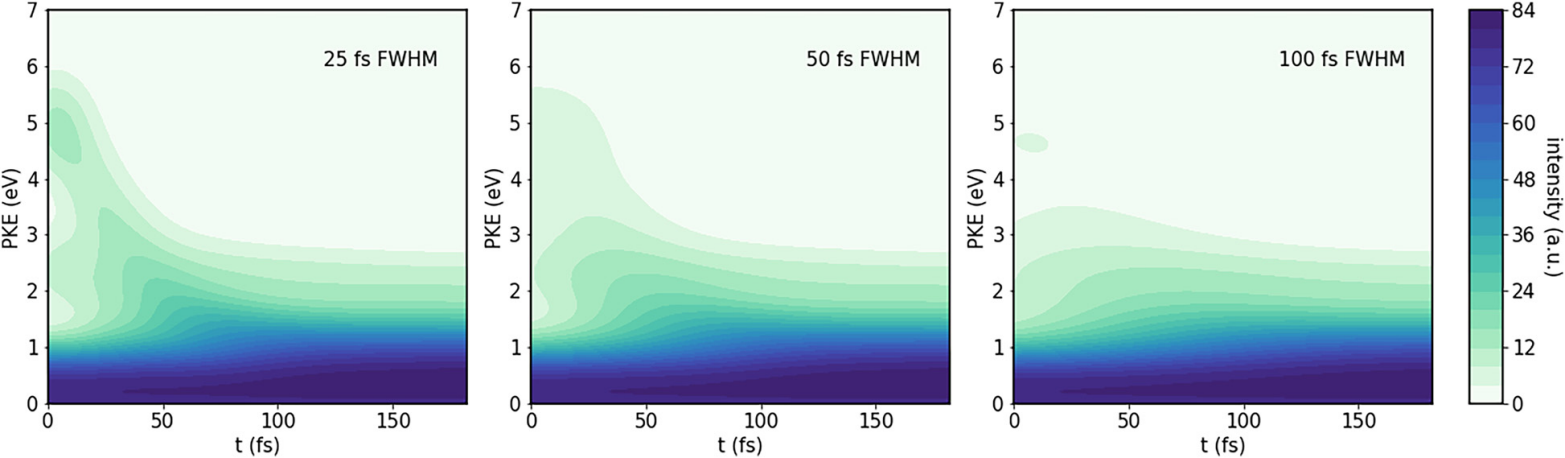
Predicted TRPES for a 133-nm probe, for different full-width at half maxima (FWHM) of the cross-correlation function.

**FIG. 9.**
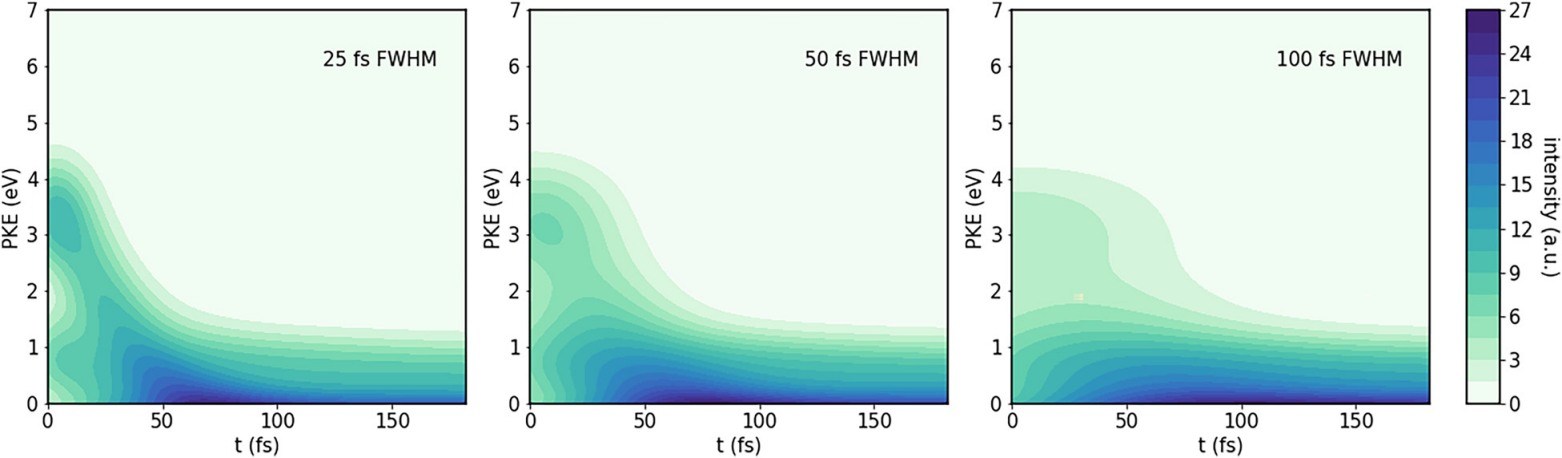
Predicted TRPES for 160 nm probe, for different FWHM of the cross-correlation function.

Adding in all of the expected sources of photoelectrons, and convoluting as described in the methods section, results in the time-resolved photoelectron spectra shown in Figs. 8 and 9. For this, we assumed a signal:direct ionization background:time zero feature ratio of 1.3:5:50. Additionally, we assumed that after the conical intersection, the ratio of population in the upper and lower spin–orbit states is 0.3:1.

As expected, using the 133-nm probe results in a lot of background in the lower-energy part of the spectrum. Furthermore, these predicted spectra show the effect of the finite pulse width of pump and probe. For cross-correlation times of 100 fs, the time-zero features dominate at early times, their width being similar to the cross-correlation full-width at half maximum (FWHM), centered at zero delay. By the time they have died out, the molecule has dissociated so far that the PKE signal does not change much anymore. In contrast, at a cross-correlation FWHM of 25 fs, the time zero features have mostly vanished within about 12.5 fs, leaving time for the dynamical feature to be visible. This highlights the importance of shorter pulse lengths, needed in order to resolve the dynamics.

### D. Time-resolved photoelectron circular dichroism

**FIG. 10.**
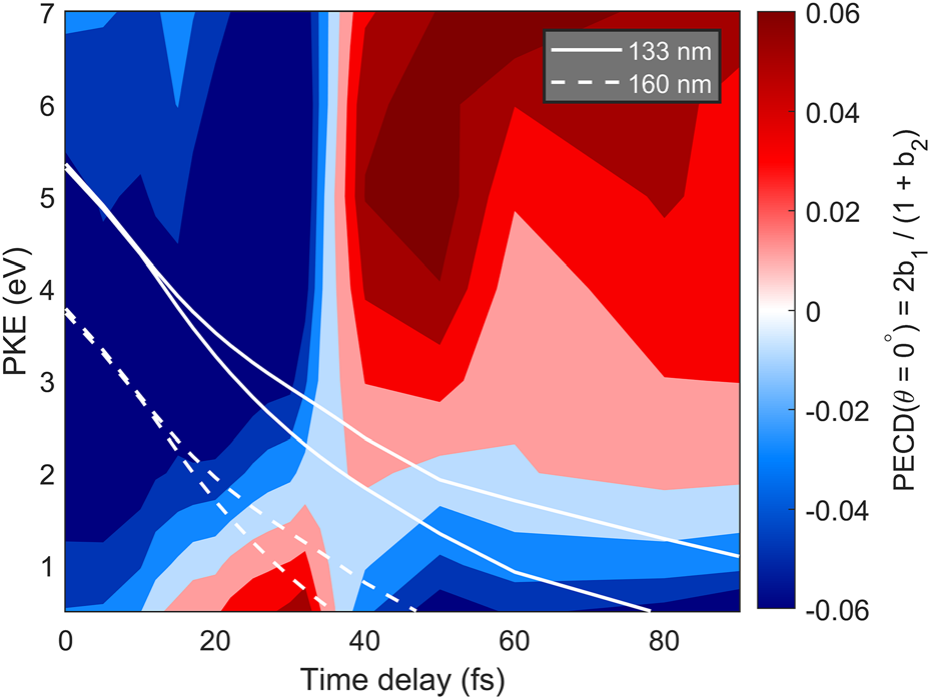
The PECD map of excited 1-iodo-2-methylbutane (conformer 1, see below) vs. photoelectron kinetic energy (PKE) and time delay after excitation. PECD is expressed as PECD $\left(\theta =0{}^{\circ})=2b_{1}/(1+b_{2}\right)$. PKE of electrons ionized with 133 nm (solid lines) and 160 nm (dashed lines) are overlayed over the PECD map. In each case, the two lines correspond to the two spin–orbit dissociation limits.

The PECD-map, which is the PECD of the excited state as a function of time and photoelectron kinetic energy, is shown in Fig. 10. Here, and in what follows, the PECD is expressed in terms of the forward–backward asymmetry along the light propagation direction, i.e., PECD( 0°) = 2*b*_1_/(1 + *b*_2_). It can be noted that the PECD varies rapidly over the course of the dissociation, and most notably flips sign at least twice, i.e., around 20–35 fs and again around 40–60 fs. In the case of CHBrFI, our recent analysis suggested scattering off the dissociating iodine atom to be an important contribution to inversions of the PECD.^24^ The observation of similar features in the present case suggests that photoelectron diffraction on iodine might indeed be the cause of the observed sign reversals.

These calculations indicate that the wavelength of the probe laser has a significant influence on the observed PECD signal and, in particular, its sign reversals. This becomes obvious from the comparison of the photoelectron kinetic energy corresponding to the two investigated wavelengths (full and dashed white lines in Fig. 10). The photoelectrons emitted following ionization with the 133-nm probe pulse will correspond to a local minimum with a negative value of the PECD (around 25 fs), at longer delays they correspond to a local maximum with positive PECD (around 40 fs), before again dropping to a negative value. In contrast, the curve corresponding to the photoelectron energies when ionizing with the 160-nm pulses leaves the region of minimal PECD earlier (around 15 fs), then reaches the region of positive PECD, but barely explores the subsequent region of negative PECD. This highlights the advantage of using the shorter-wavelength 133-nm probe pulses, which should enable the observation of at least two sign reversals, and probably more beyond the region covered by the present calculations. Even though a larger static background will be present, a larger TRPECD effect should also be measured because the static background can be subtracted,^24^ thereby mitigating the problem it poses in the experiment.

### E. The role of other conformers

**FIG. 11.**
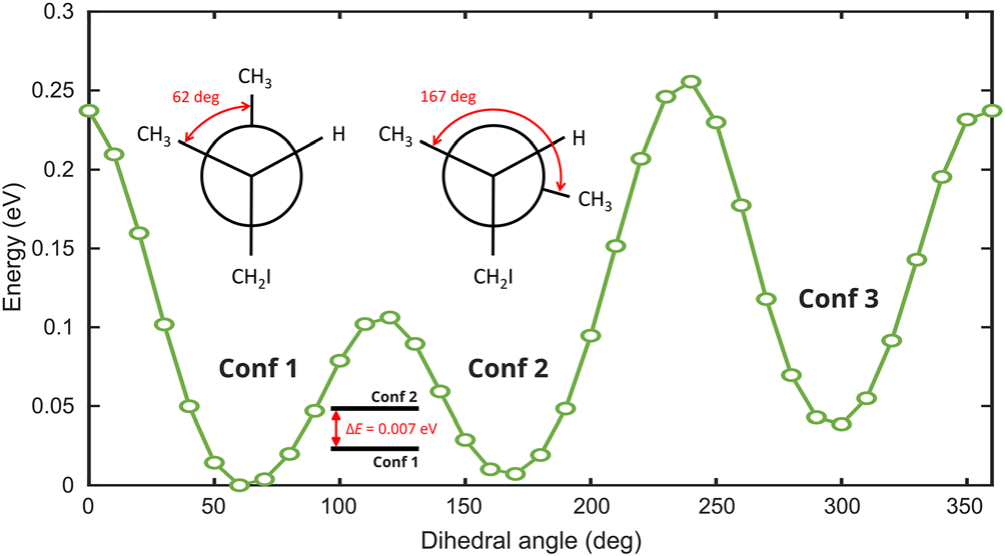
Conformation scan at MP2/cc-pVTZ level of theory. Conformers are formed by a rotation around the C_2_–C_3_ bond. Altogether three conformers (Conf 1, Conf 2, and Conf 3) exist. Two conformers (1 and 2) are nearly degenerate with an energy splitting ΔE=0.007 eV. These conformers are shown with their Newman projections.

All calculations presented so far were performed on the most stable conformer of 1-iodo-2-methylbutane. An exploration of the potential energy surface of this molecule as a function of the dihedral angle between the methyl groups in 2- and in terminal position (Fig. 11) reveals the presence of two additional conformers, one of which (conformer 2) lies only 7 meV above the most stable one, whereas conformer 3 lies ∼40 meV higher. In a typical supersonic expansion, therefore, it should be possible to depopulate conformer 3, but it will be difficult to obtain conformer 1 as a pure sample. At an internal temperature of 30 K, the population ratio of conformer 1:conformer 2 will be ∼2.8:1.

**FIG. 12.**
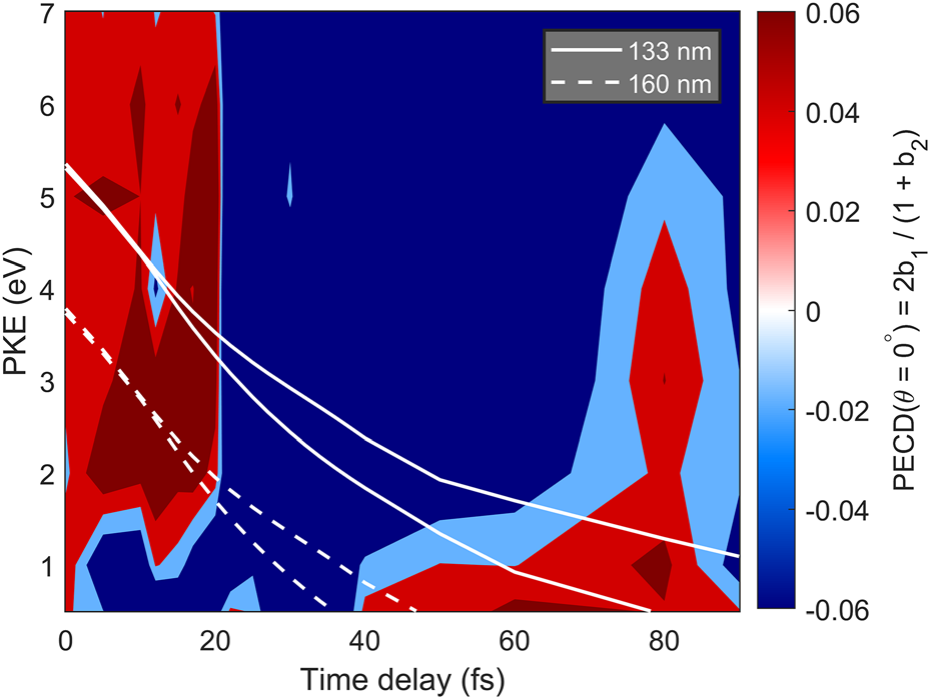
The PECD map of excited 1-iodo-2-methylbutane (conformer 2) vs. photoelectron kinetic energy (PKE) and time delay after excitation. The PECD is expressed as PECD $\left(\theta =0{}^{\circ})=2b_{1}/(1+b_{2}\right)$. The PKE of electrons ionized with 133 nm (solid line) and 160 nm (dashed line) are overlayed over the PECD map. In each case, the two lines correspond to the two spin–orbit dissociation limits.

Since it will be challenging to entirely suppress the population of conformer 2, we have calculated the PECD map for conformer 2 as well, which is shown in Fig. 12 and ought to be compared to that of conformer 1 in Fig. 10. The similarities between the two PECD maps are striking. Up to an overall opposite sign, the two PECD maps are remarkably similar. The nearly vertical region of the first sign flip occurs at ∼35 fs in conformer 1, as compared to ∼20 fs in conformer 2. Below kinetic energies of ∼1.5 eV, the PECDs have the opposite signs compared to the region above kinetic energies of ∼1.5 eV, and this holds also for both conformers. The main difference between the two conformers is found around 80 fs, where a region of positive PECD extends up to kinetic energies of ∼5 eV in conformer 2, but such a feature is not observed in conformer 1.

The time-resolved PECDs of the two conformers are, thus, surprisingly similar up to an overall factor of −1. This raises the question why these two conformers behave almost as enantiomers of each other. This can be understood by rotating the Newmann projection of conformer 2 in Fig. 11 counterclockwise by 120° and recalling that we are discussing the PECD of photoelectrons emitted from the 2-methyl-butyl radical once the C–I bond can be considered to be broken, because 133-nm light does not ionize atomic iodine. In the absence of the iodine atom, the 2-methyl-butyl radicals produced from conformer 1 and 2 are almost enantiomers of each other, up to one missing hydrogen atom. This explains why the calculated TRPECDs of the two conformers are almost opposite of each other. We note that a very similar result has been obtained for calculated TRPECDs of I(4d) of the same molecule.^32^

The existence and energetic proximity of conformer 2 of 1-iodo-2-methylbutane, thus, represents a non-negligible challenge for the realization of TRPECD with this molecule. Unless conformer 2 can be efficiently depopulated in a superonic expansion or by active conformer selection, its presence will tend to reduce the observed TRPECD. Such challenges have also been reported in the context of the recent experimental work at FLASH.^32^

## IV. CONCLUSIONS

In this work, we have developed a calculation protocol to predict TRPECD effects and have applied it to 1-iodo-2-methylbutane. This method enables the prediction of TRPECD during photo-induced reactions, such as photodissociation, as demonstrated in the present work. Our method relies on the calculation of the chiral *b*_1_ parameter, which encodes the molecular chirality into the photoelectron angular distribution. We have shown how to extract the value of the *b*_1_ parameter from the photoionization matrix elements, which in turn are obtained from quantum-scattering calculations. We have calculated TRPECDs for a broad range of molecular structures describing the course of a photo-induced reaction, which are obtained from *ab initio* molecular-dynamics simulations. Since the PECD parameter for a certain structure is energy-dependent, we need to know the kinetic energy of the electron that is ejected upon absorption of the probe photon. This energy was extracted from electronic structure calculations of the neutral and cationic potential-energy surfaces. In addition to the TRPECD, we thereby also obtained a prediction for the time-resolved photoelectron spectra.

We demonstrated our method on 1-iodo-2-methylbutane, which can be photodissociated with commonly available 266-nm pump pulses and is, therefore, a possible candidate for TRPECD experiments. The simulations performed in this work have revealed the influence on the temporal resolution and the probe wavelength on the experimental results. We have found a rapid variation of the PECD parameters over the first tens of femtoseconds of the dissociation reaction, including at least two sign reversals of the PECD parameter. These results agree well with recent experimental observations and their theoretical interpretation in terms of photoelectron diffraction.^24^ The specific challenges introduced by the presence of conformers of 1-iodo-2-methylbutan have been identified and discussed. Looking forward, our method can be applied to describe TRPECD experiments of the broad class of chiral molecules, offering a promising tool for the interpretation of ultrafast TRPECD experiments on chiral photoreactions.

## Data Availability

The data that support the findings of this study are available within the article.
